# Correlation of diffusion MRI with the Ki-67 index in non-small cell lung cancer

**DOI:** 10.1515/raon-2015-0032

**Published:** 2015-08-21

**Authors:** Adem Karaman, Irmak Durur-Subasi, Fatih Alper, Omer Araz, Mahmut Subasi, Elif Demirci, Mevlut Albayrak, Gökhan Polat, Metin Akgun, Nevzat Karabulut

**Affiliations:** 1 Department of Radiology, Ataturk University, Medical Faculty, Erzurum, Turkey; 2 Department of Pulmonary Diseases, Ataturk University, Medical Faculty, Erzurum, Turkey; 3 Department of Thoracic Surgery, Erzurum Regional Training and Research Hospital, Erzurum, Turkey; 4 Department of Pathology, Ataturk University, Medical Faculty, Erzurum, Turkey; 5 Department of Radiology, Pamukkale University, Medical Faculty, Denizli, Turkey

**Keywords:** diffusion weighted-magnetic resonance imaging, apparent diffusion coefficient, Ki-67 index, adenocarcinoma, squamous cell carcinoma

## Abstract

**Background:**

The primary objective of the study was to evaluate the association between the minimum apparent diffusion coefficient (ADC_min_) and Ki-67, an index for cellular proliferation, in non-small cell lung cancers. Also, we aimed to assess whether ADC_min_ values differ between tumour subtypes and tissue sampling method.

**Methods:**

The patients who had diffusion weighted magnetic resonance imaging (DW-MRI) were enrolled retrospectively. The correlation between ADC_min_ and the Ki-67 index was evaluated.

**Results:**

Ninety three patients, with a mean age 65 ± 11 years, with histopathologically proven adenocarcinoma and squamous cell carcinoma of the lungs and had technically successful DW-MRI were included in the study. The numbers of tumour subtypes were 47 for adenocarcinoma and 46 for squamous cell carcinoma. There was a good negative correlation between ADC_min_ values and the Ki-67 proliferation index (r = −0.837, p < 0.001). The mean ADC_min_ value was higher and the mean Ki-67 index was lower in adenocarcinomas compared to squamous cell carcinoma (p < 0.0001). There was no statistical difference between tissue sampling methods.

**Conclusions:**

Because ADC_min_ shows a good but negative correlation with Ki-67 index, it provides an opportunity to evaluate tumours and their aggressiveness and may be helpful in the differentiation of subtypes non-invasively.

## Introduction

Diffusion weighted magnetic resonance imaging (DW-MRI) is a promising MRI technique used in the evaluation of lung tumours. It has been increasingly used for the detection, differential diagnosis and evaluation of tumour characteristics, including grading and prediction of the therapeutic response.^1^–^7^ DW-MRI is a functional imaging technique that reveals physiological information by quantifying the diffusion of water molecules in tissues. The extent of this diffusion is measured using the apparent diffusion coefficient (ADC). Malignant tissues tend to have a lower ADC and demonstrate higher signal intensity on a DW-MRI image due to their increased cellularity and larger nuclei with abundant macromolecular proteins.^8^,^9^

The Ki-67 protein (also known as MKI67) is a cellular proliferation marker. During interphase, the Ki-67 antigen can only be detected within the cell nucleus; however, in mitosis, most of the Ki-67 is relocated to the surface of the chromosomes. Ki-67 protein is present during all active phases of the cell cycle (G1, S, G2, and mitosis), but is absent in resting cells (G0). The Ki-67 proliferation index, one of the biological markers used in histopathological evaluation, is an important criterion in the differentiation of benign and malignant tumours.^10^–^12^ It is also correlated with the clinical course of cancer and has been shown to have prognostic value for treatment response, tumour recurrence and survival in brain, breast, bladder and prostate tumours, meningioma and nephroblastoma.^13^–^19^ The Ki-67 index has also been used routinely in the evaluation of lung tumours and has been shown to be an important prognostic factor for lung cancer.^3^,^6^,^20^–^27^ Although a few studies have evaluated the association of ADC with Ki-67 index in lung tumours^3^,^6^, no study has previously investigated differences in the ADC/Ki 67 correlation in different tumour subtypes.

In this study, our primary objective was to evaluate whether there is an association between the minimum ADC (ADC_min_), determined on DWMRI, and Ki-67, which is a cellular proliferative index. Our secondary aim was to assess whether ADC_min_ values differ between the adenocarcinomas and squamous cell carcinomas of the lungs and also differ according to the pathologic sampling method used, surgical excision specimen and biopsied material.

## Methods

### Study population

Between January 2012 and December 2013, records for 104 consecutive patients with histopathologically proven primary adenocarcinoma and squamous cell carcinoma of the lungs, and who had technically successful images on DW-MRI were retrieved from the hospital’s pathology database. The patients who were previously treated (n = 5) and\or had an interval of more than 15 days between DW-MRI and biopsy (n = 6) were excluded from the study. All measurements, including calculation of Ki-67 index and ADC_min_ values, were done in the same lesion for each patient. The protocol of the retrospective study was approved by the institutional ethics committee and the requirement for informed consent was waived.

### Imaging technique, DW-MRI

It was performed with a 3 tesla scanner (MAGNETOM Skyra, Siemens Healthcare, Erlangen, Germany). Conventional MRI included an axial T1-weighted sequence (repetition time, 104 ms; echo time, 4.92 ms; 1 excitation) and an axial T2-weighted sequence (repetition time, 1400 ms; echo time, 101 ms; 1 excitation). Breath-free DW-MRI was performed in the axial plane using a single-shot, spin-echo echo-planar imaging sequence with the following parameters: repetition time, 6500 ms; echo time, 61 ms; real spatial resolution in the phase-encoding direction, 3.7 mm; flip angle, 90°; diffusion gradient encoding in three orthogonal directions; b value b = 50, b = 400 and b = 800 s/mm^2^; field of view, 380 mm × 380 mm × 310 mm; matrix size, 113 × 192; slice thickness, 6 mm; section gap, 0 mm; 2 signals acquired.

### Image analysis

**FIGURE 1.**
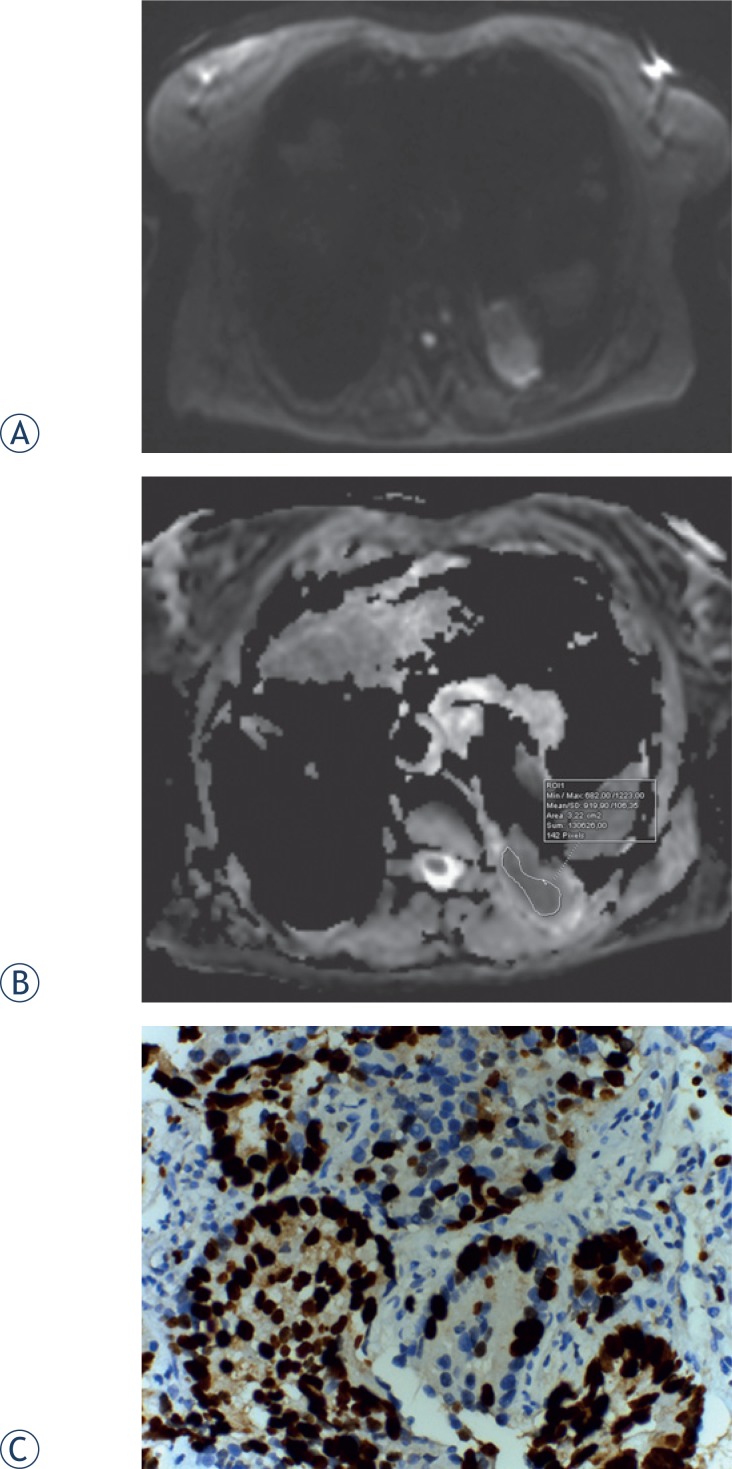
Diffusion-weighted (DW)-MRI, apparent diffusion coefficient (ADC) map of a 62-year-old female with adenocarcinoma. **(A)** Tumour shows heterogeneously high signal intensity on DW-MRI, for which the b value is 800 s/mm^2^. **(B)** On the ADC map, the tumour demonstrates heterogeneous diffusion restriction. **(C)** Proli ferative index 95% in glandular epithelium (Ki-67X400).

We analysed the lesions using DW-MRI images in association with T1- and T2-weighted images in order to identify accurately. The ADC of the tumour was then calculated to quantitatively analyse the degree of diffusion, using the following formula: ADC = −ln(S/S_0_) / (b−b_0_), where S_0_ and S are the signal intensities, obtained at three different diffusion gradients (b = 50, b = 400 and b = 800 s/mm^2^). The ADC maps were reconstructed at a workstation. While establishing the size and region for the ROI, positioning in the larger area was considered in order to minimize the effect of region on hemodynamic inhomogeneity of tumour by avoiding necrotic, cystic or calcific areas by referring to T2 and T1-weighted images.^28^,^29^ The ADC_min_ values within the ROI were then used in statistical analyses (Figure 1). In analyses workstation (Syngo Via Console, software version 2.0, Siemens AG Medical Solutions, Erlangen, Germany) was used.

### Calculation of Ki-67 index

Archived paraffin blocks belonging to the patients were transferred to polylysine glass slides with 4-micron thick sections. Immunochemistry was performed using a Lecia Bond-max automated immunostainer (Leica Microsystems, Newcastle, UK), as described manufacturers protocol. For Ki-67 staining, Ki-67 antibody (NCL-L-Ki67-MM1, monoclonal, 1:60, Novocostra, Newcastle, UK) was used. The sections prepared for examination were evaluated by two pathologists who were blinded to each-other. Firstly, ten areas having highest expression of Ki-67 were determined at low magnification. Then, these areas were further analysed at a single high power field (400 × magnification). Ki-67 expression was defined as the percent of Ki-67-positive tumour cells divided by the total number of tumour cell within one high power field.^26^,^30^ In the last step, Ki-67 index was calculated as the average percentage of those fields.

### Statistical Analysis

Analyses were performed using IBM SPSS 20.0 for Mac software. The correlation between ADC_min_ and the Ki-67 index was evaluated using Spearman’s correlation coefficient. Mann-Whitney U tests were used to assess the difference between the ADC_min_ and the Ki-67 index for the different tumour subtypes. A p value of less than 0.05 was considered statistically significant.

## Results

Ninety three patients, with a mean age 65 ± 11 years ranged between 43 and 84, with histopathologically proven primary adenocarcinoma (n = 47) and squamous cell carcinoma (n = 46) of the lungs and had technically successful DW-MRI were included in the study. Histopathological diagnoses were obtained through transthoracic or transbronchial biopsy in 65 subjects and 28 patients underwent surgery.

The mean ADC_min_ value for all the lesions was 0.77 ± 0.15 × 10^−3^ mm^2^/sec (range, 0.50–1.00 × 10^−3^ mm^2^/sec). The mean ADC_min_ value for adenocarcinomas was 0.83 ± 0.12 × 10^−3^ mm^2^/sec and that of squamous cell carcinomas was 0.70 ± 0.16 × 10^−3^ mm^2^/sec; there was a significant difference between these values (p < 0.0001). The mean Ki-67 was 43.5 ± 22.2 for all the tumours (range, 5–96), with a mean of 30.8 ± 14.1 for adenocarcinomas and 55.9 ± 21.8 for squamous cell carcinoma; the difference between tumour subtypes was significant (p < 0.0001).

**FIGURE 2.**
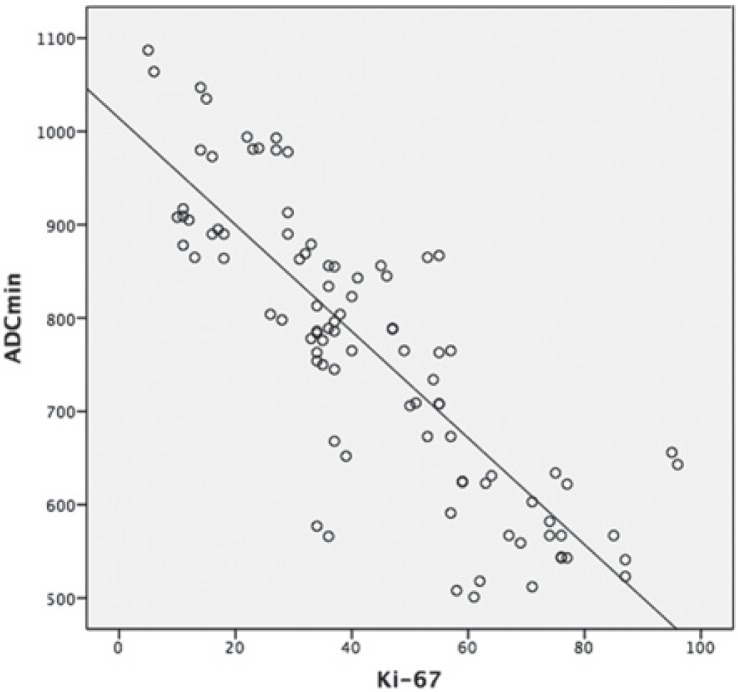
The graph shows a negative correlation between the minimum apparent diffusion coefficient (ADC_min_) and the Ki-67 index in lung tumours (r = −0.837, p < 0.001).

**FIGURE 3.**
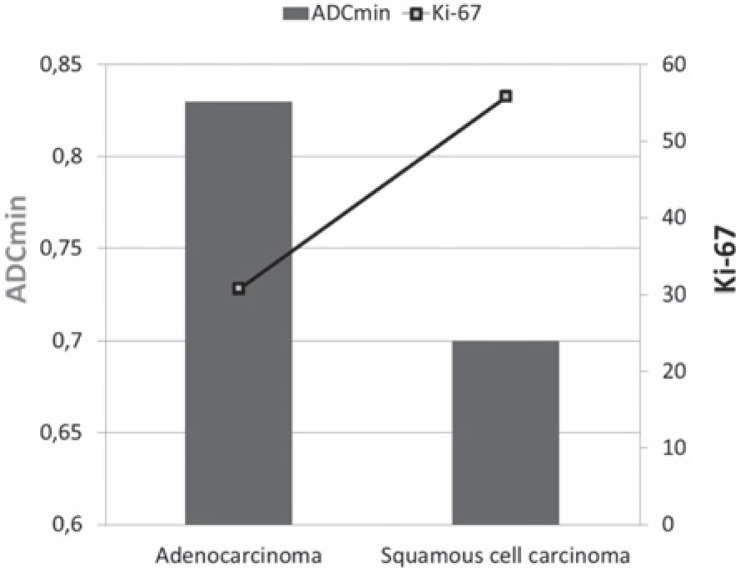
The graph shows average minimum apparent diffusion coefficient (ADC_min_) values for adenocarcinoma and squamous cell carcinoma according to Ki-67 index. Bars are for ADC_min_ values and line is for Ki-67.

There was a negative correlation between ADC_min_ values and the Ki-67 proliferation index (p < 0.001, r = −0.837) (Figure 2). The ADC_min_ values were lower in the cases with higher Ki-67 grades. The mean ADC_min_ values and Ki-67 index for adenocarcinomas and squamous cell carcinomas of the lung are shown in Figure 3. There was no statistical difference of Ki-67 and ADC_min_ values between biopsied material and surgical specimen. The mean Ki-67 was 45.3 ± 22.8 vs 39.3 ± 19.8 and the mean ADC_min_ value was 0.76 ± 0.16×10^−3^ vs 0.78 ± 0.14 × 10^−3^ for biopsied material and surgical specimen, respectively. In the comparative evaluation of correlation between ADC_min_ and the Ki-67 proliferation index that measured either in surgical specimen or biopsied material, the Ki-67 index of surgical specimens was slightly better correlated with ADC_min_ values without statistical significance (r = −0.870 vs. −0.617) compared to biopsied material.

## Discussion

Our results showed that there is a negative correlation between the ADC_min_ and the Ki-67 index of lung cancers, which reflects aggressiveness of a tumour. ADC_min_ values for adenocarcinomas were higher than those for squamous cell carcinomas. This finding indicates that ADC_min_ may have a role in discriminating adenocarcinomas from squamous cell carcinomas, as well as being used for evaluating the aggressiveness of the tumour. Also, a low ADC_min_ value can potentially be used as a non-invasive surrogate biomarker for the Ki-67 index for the evaluation of lung tumour characteristics, regardless of subtype.

Lung cancer is the leading cause of cancer-related deaths.^31^ Until now, the Ki-67 proliferation index, reflecting aggressiveness of a tumour has been used to determine the prognosis. Malignant tumours are characterized by increased Ki-67 proliferation index due to their cellularity, larger nuclei with more abundant macromolecular proteins, a larger nuclear/cytoplasmic ratio and less extracellular space relative to normal tissue. As these characteristics also restrict the diffusion of water molecules, ADC_min_ decreases in malignant tumours.^8^,^9^,^32^

Because ADC_min_ is found to have stronger correlation with Ki-67 index compared to ADC_mean_, we used ADC_min_ in our study.^15^ Apparent diffusion coefficient can be used in the non-invasive assessment of suspicious masses, for example, to differentiate metastatic lymph nodes from those that are benign when they cannot be differentiated by size criteria.^5^ ADC values also correlate with tumour grades.^4^,^17^,^18^ Recent studies have shown that ADC may be more useful than FDG-PET in the differentiation of malignant tumours from benign lesions^3^,^6^ and the new approaches using PET\MRI may provide more promising results in the future.^33^ Among primary lung cancers, ADC values are usually low in cases with small cell carcinomas, but the values for adenocarcinomas and squamous cell carcinomas are usually similar.^3^,^4^ However Matoba *et al.* stated that ADCs of well-differentiated adenocarcinoma appear to be higher than those of other histologic lung carcinoma types.^23^ Our findings demonstrate that adenocarcinomas showed higher ADC values than squamous cell carcinomas, and had weaker staining diffusivity and intensity of Ki-67.

A high Ki-67 and low ADC_min_ value indicates that a tumour has a high proliferation rate. Ki-67 values obtained using an invasive method reflect only the level in the sampled tissue; this is a particular problem when using biopsy. Since lung carcinomas are not always homogenous, the biopsy site can influence the results. This could be reflected in the fact that in our study the correlation between ADC_min_ and Ki-67 proliferation index was stronger for surgical than for biopsy samples. Unlike these invasive sampling methods, ADC_min_ values obtained by DW-MRI in a non-invasive manner can be calculated from anywhere in the tumour, providing an entire and reproducible assessment of the tumour. Furthermore, since the region with the lowest ADC_min_ value is likely to be the most aggressive portion.^17^,^34^ DWI could also help in the selection of an appropriate biopsy site within the tumour.

An association between the ADC value and the Ki-67 index has been shown for various kinds of tumours^2^,^14^–^18^,^34^–^38^, including lung cancer.^3^,^6^ Wang *et al.*, in their study on DWI in pancreatic endocrine tumours, reported a correlation coefficient of −0.70^2^, while Onishi *et al.* reported a correlation coefficient of −0.825 for mucinous breast carcinoma.^15^

Previous studies reporting ADC values of lung carcinoma have been conducted under various magnet strengths, and reported ADC values are lower in magnets with a stronger field. Matoba *et al.* reported mean ADC values of 1.63 × 10^−3^ mm^2^/sec ± 0.5 (mean ± SD) for squamous cell carcinomas, 2.12 × 10^−3^ mm^2^/sec ± 0.6 for adenocarcinomas, 1.30 × 10^−3^ mm^2^/sec ± 0.4 for large-cell carcinomas, and 2.09 × 10^−3^ mm^2^/sec ± 0.3 for small-cell carcinomas, using a 1.5 T scanner. Usuda *et al.*^6^ found that malignant nodules had a mean ADC of 1.27 ± 0.35 ×10^−3^ mm^2^/sec on a 1.5T system. Using a 3.0 T scanner, Zhang *et al.* reported that malignant pulmonary nodules had a mean ADC of 0.87 ± 0.16 × 10^−3^ mm^2^/ sec. Similarly, we found a mean ADC_min_ of 0.77 ± 0.12 × 10^−3^ mm^2^/sec in our study conducted on a 3.0 T scanner. These values are lower than those were reported by the studies conducted using 1.5 T systems.^6^,^23^ However, Kivrak *et al.* noted that ADC values vary for different MRI systems with the same magnetic field strength (1.5 T).^39^ On the other hand, some authors reported that ADC values might not change for different organ systems under different magnetic fields.^40^ However, they only used healthy volunteers and neither pathologic conditions nor image quality was not assessed. Further work is still needed to investigate the effect of magnetic field strength on the ADC of different organ systems.

One of the strongest side of our study was that we used 3 tesla MRI, which has increased signal to noise ratio, spatial resolution, temporal resolution, etc. Thus, decreased imaging time increased patients’ cooperation and we had better qualified images. Our study had a few limitations. Our study population was relatively small and, although our results are robust, prospective studies with larger series are warranted to confirm our results. Additionally, to be able to generalize our results to all subtypes of lung cancer, such as small cell carcinomas and the other subtypes of non-small cell lung cancer, which we had very limited number of such cases during the study period, need to be included in future studies. Because we had no data about survival of the cases, we could not conclude any association between ADC_min_ or Ki-67 and survival. However, use of ADC_min_ may provide new insight to the evaluation of lung cancer including benign-malignant discrimination, the possibility of evaluation all lesions and lymph nodes noninvasively, even in the cases that tissue sampling is difficult, as well as predicting the prognosis of tumour by using it as a surrogate marker of Ki-67 index.

In conclusion, our results suggested that ADC_min_ values were inversely correlated with Ki-67 index in non-small cell lung cancer and may be used as a surrogate marker of Ki-67 index in the evaluation of tumour aggressiveness with the advantage of its non-invasiveness and without requirement of tissue sampling of all the lesions.
